# Critical role of SOX2–IGF2 signaling in aggressiveness of bladder cancer

**DOI:** 10.1038/s41598-020-65006-z

**Published:** 2020-05-19

**Authors:** Yu-Fan Chiu, Chia-Chang Wu, Ming-Han Kuo, Chia-Cheng Miao, Ming-Yi Zheng, Pei-Yu Chen, Sheng-Chieh Lin, Junn-Liang Chang, Yuan-Hung Wang, Yu-Ting Chou

**Affiliations:** 1 Institute of Biotechnology, College of Life Science, National Tsing Hua University, Hsinchu, Taiwan; 2 Department of Urology, Shuang Ho Hospital, Taipei Medical University, New Taipei City, Taiwan; 3 Department of Medical Research, Shuang Ho Hospital, New Taipei City, Taiwan; 4 Department of Urology, School of Medicine, College of Medicine, and TMU Research Center of Urology and Kidney (TMU-RCUK), Taipei Medical University, Taipei, Taiwan; 5 Graduate Institute of Clinical Medicine, College of Medicine, Taipei Medical University, Taipei, Taiwan; 6 Graduate Institute of Integrated Medicine, China Medical University, Taichung, Taiwan; 7 Department of Pathology and Laboratory Medicine, Taoyuan Armed Forces General Hospital, Taoyuan, Taiwan; 8 Department of Biomedical Engineering, Ming Chuan University, Taoyuan, Taiwan

**Keywords:** Bladder cancer, Growth factor signalling

## Abstract

Signaling elicited by the stem cell factors SOX2, OCT4, KLF4, and MYC not only mediates reprogramming of differentiated cells to pluripotency but has also been correlated with tumor malignancy. In this study, we found *SOX2* expression signifies poor recurrence-free survival and correlates with advanced pathological grade in bladder cancer. *SOX2* silencing attenuated bladder cancer cell growth, while its expression promoted cancer cell survival and proliferation. Under low-serum stress, *SOX2* expression promoted AKT phosphorylation and bladder cancer cells’ spheroid-forming capability. Furthermore, pharmacological inhibition of AKT phosphorylation, using MK2206, inhibited the *SOX2*-mediated spheroid formation of bladder cancer cells. Gene expression profiling showed that *SOX2* expression, in turn, induced *IGF2* expression, while *SOX2* silencing inhibited *IGF2* expression. Moreover, knocking down *IGF2* and *IGF1R* diminished bladder cancer cell growth. Lastly, pharmacological inhibition of IGF1R, using linsitinib, also inhibited the *SOX2*-mediated spheroid formation of bladder cancer cells under low-serum stress. Our findings indicate the SOX2–IGF2 signaling affects the aggressiveness of bladder cancer cell growth. This signaling could be a promising biomarker and therapeutic target for bladder cancer intervention.

## Introduction

Bladder cancer arises from the urinary bladder’s epithelial lining, called the urothelium, and is one of the most common urinary system malignancies^1^. Most patients are diagnosed with non-invasive bladder cancer, and surgery is the typical treatment option^2^. However, just over half of these patients will experience tumor recurrence^3^. Chemotherapy is the conventional treatment for patients with advanced stage bladder cancer, but few can be cured^4^. Therefore, novel biomarkers for monitoring, and therapeutic targets for targeting, bladder cancer progression are urgently needed.

Transcription factors involved in embryonic stem cell (ESC) and induced pluripotent stem cell (iPSC) signaling, such as SOX2, OCT4, MYC, and KLF4, have been linked to tumor progression in various cancers^5,6^. Among them, SOX2 is essential to self-renewal and differentiation of several adult tissue progenitor cells^7–9^. SOX2 expression has been implicated in human breast and lung cancers^10–12^. Genomic amplification of *SOX2* has been observed in lung squamous cell carcinoma^13^, while genomic amplification of *SOX4*, another SOX family member, has been reported in bladder cancer^14,15^. SOX2 promotes abnormal proliferation of lung cancer cells and controls tumor initiation in skin squamous cell carcinoma^16,17^. Although there is high SOX2 expression in bladder cancer^18^, the oncogenic mechanism underlying SOX2-mediated tumor malignancy remains unclear.

Insulin-like growth factor 2 (IGF2), a mitogenic peptide hormone, is highly expressed during embryonic development^19^, and overexpressed in tumors associated with more aggressive status^20^. Previous studies suggest that IGF2 binds to its receptor, IGF1R, to initiate tumorigenesis of breast and lung cancers^21,22^ and promote progression of endometrial and gastric cancers^23,24^. Loss of imprinting contributes to overexpression of IGF2 in cancers of the prostate and colon and cancers with stem cell-like features^25–28^, while deregulation of IGF2 in cancers is also attributable to abnormal expression of transcription factors^29–31^. Insulin-like growth factor-binding protein 1 (IGFBP1) is a secreted protein serving as a negative regulator that competes with IGF ligands, thus preventing ligand–receptor activation^32–34^. Although IGF2/IGF1R signaling enhances tumor progression in several cancers, it is unclear whether IGF2/IGF1R signaling contributes to bladder cancer progression.

In this study, we found SOX2 is a prognostic marker in bladder cancer patients, signifying poor survival. We also found SOX2 promotes AKT phosphorylation in bladder cancer cells by inducing *IGF2* and *IGF1R* expression and suppressing *IGFBP1*. We further characterized the potential of *IGF1R* signaling as a biomarker and therapeutic target in treating bladder cancer.

## Results

### SOX2 expression is correlated with tumor malignancy in bladder cancer

**Figure 1:**
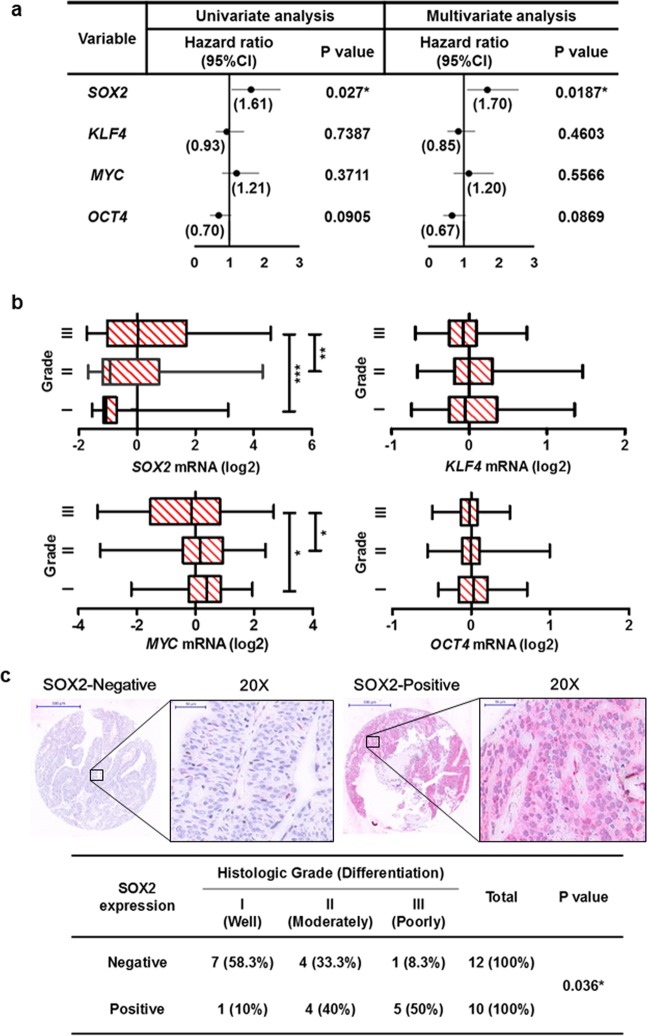
*SOX2* is associated with poor histologic differentiation of bladder cancer. (**a**) Univariate and multivariate analyses for recurrence-free survival based on the expression of stem cell factors *SOX2*, *KLF4*, *MYC*, and *OCT4* in bladder cancer patients from GSE32894 database. *P < 0.05. (**b**) Gene expression analysis to assess *SOX2*, *KLF4*, *MYC*, and *OCT4* levels and their correlation with histologic grade of bladder tumors from GSE32894 database. One Way ANOVA and Tukey’s multiple comparison analysis were used to determine statistical significance: *P < 0.05; **P < 0.01; ***P < 0.001. (**c**) Representative images (upper) of immunohistochemical analysis for SOX2-negative and SOX2-positive staining in bladder transitional cell carcinoma. Scale bars: 500 μm (whole section); 50 μm (inset, 20× magnification). Fisher’s exact test (lower) for association between SOX2 expression and tumor grade in bladder transitional cell carcinoma (n = 22).

Because factors in ESC signaling and iPSC reprogramming have been linked to tumor malignancy, we used the Cox’s proportional hazards model to analyze the link between *SOX2*, *KLF4*, *MYC* and *OCT4* expression and recurrence-free survival outcome for bladder cancer patients (Fig. 1a). Both univariate and multivariate regression analyses revealed that only *SOX2* expression correlated with poor recurrence-free survival (Fig. 1a, and Supplementary Table 1). Box-and-whisker plots showed that *SOX2* expression was also associated with advanced tumor grade of bladder cancer (Fig. 1b). Immunohistochemistry was used to verify SOX2 expression in primary bladder tumors, which showed SOX2 expression was high in tumors with “poorly differentiated” malignant grade (Fig. 1c). These data highlight *SOX2*’s potential involvement in bladder cancer tumor malignancy.

### SOX2 regulates the growth of bladder cancer cells

**Figure 2:**
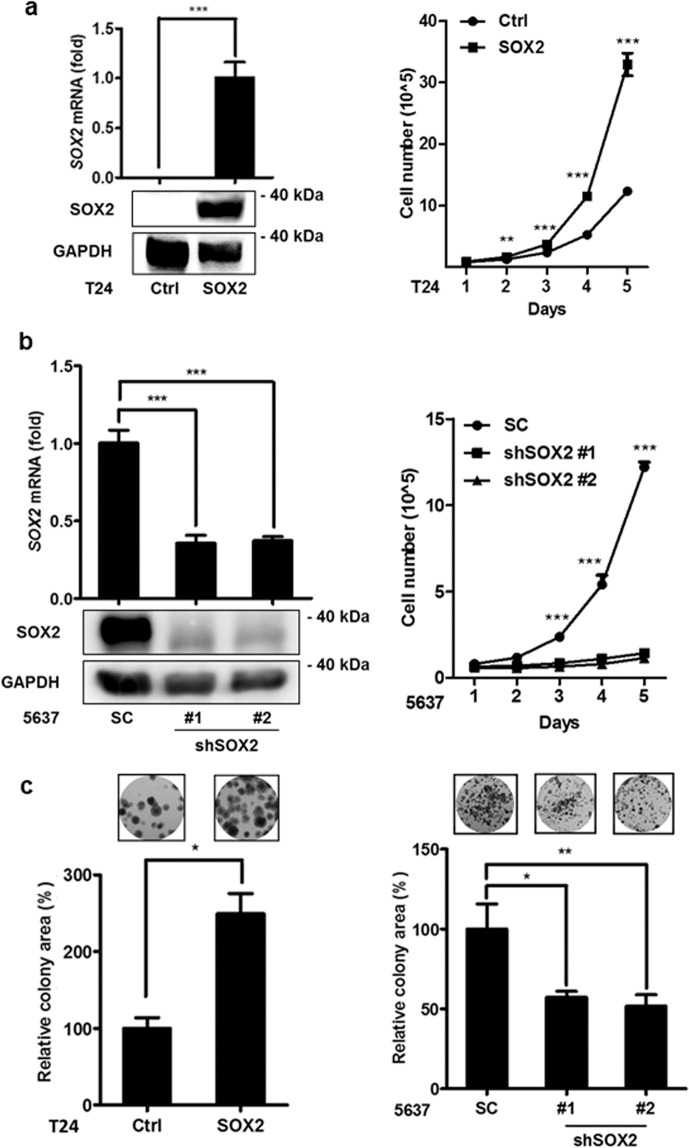
SOX2 mediates growth of bladder cancer cells. (**a**) qPCR (upper left) and immunoblotting (lower left) analysis to assess *SOX2* mRNA and protein expression, respectively, in T24 cells transduced with the lentiviral vector encoding *SOX2* cDNA (SOX2) or empty control vector (Ctrl). Trypan blue cell exclusion analysis of T24 cells transduced with the lentiviral vector encoding *SOX2* cDNA (SOX2) or empty control vector (Ctrl) for the indicated days. Results are the average of three replicates and expressed as the mean ± S.D. **P < *0.01*, ***P < *0.001*. (**b**) qPCR (upper left) and immunoblotting (lower left) analysis of *SOX2* expression in 5637 cells transduced with the lentiviral vector encoding shRNA against *SOX2* (shSOX2) or scrambled control vector (SC). Trypan blue cell exclusion analysis of 5637 cells transduced with the lentiviral vector encoding shSOX2 or scrambled control vector (SC) for the indicated days. Results are the average of three replicates and expressed as the mean ± S.D. The #1 and #2 indicate the two distinct shRNAs that target different regions within *SOX2*. ***P < *0.001*. (**c**) Clonogenic analysis (left) to assess the *SOX2* expression effect on the colony-forming ability in T24 cells transduced with the lentiviral vector encoding *SOX2* cDNA (SOX2) or empty control vector (Ctrl). Clonogenic analysis (right) to assess the *SOX2* knockdown effect on the colony-forming ability in 5637 cells transduced with the lentiviral vector encoding shSOX2 or scrambled control vector (SC). Colonies were subjected to crystal violet staining and quantified by ImageJ analysis. Results are the average of three replicates and expressed as the mean ± S.D. *P < 0.05, **P < 0.01.

Assessing *SOX2* expression in bladder cancer cell lines showed its expression was considerably lower in T24 cells than in 5637 cells (Supplementary Figure S1). To investigate its role in bladder cancer oncogenesis, *SOX2* was ectopically expressed in T24 cells using the lentiviral transduction system, and its expression was confirmed with immunoblotting and qPCR (Fig. 2a left). Trypan blue cell exclusion and alamarBlue proliferation analysis showed that *SOX2* expression promoted cell proliferation (Fig. 2a right and Supplementary Figure S2a). Because 5637 represents a bladder cancer cell line with high *SOX2* expression, we adopted the lentiviral shRNA system to knock down *SOX2* in 5637 cells to further investigate the effect of eliminating *SOX2* function. qPCR and immunoblotting assays indicated that endogenous *SOX2* mRNA expression was suppressed by sh*SOX2* (Fig. 2b left). The trypan blue cell exclusion test, alamarBlue proliferation assay, and cell cycle analysis revealed that silencing *SOX2* in 5637 cells inhibited cell proliferation due to S-phase arrest during cell cycle progression (Fig. 2b right and Supplementary Fig. S2b,c). In addition, clonogenic assays showed *SOX2’s* ectopic expression increased T24 cells’ colony-forming capability, whereas knockdown of *SOX2* in 5637 cells weakened colony formation. (Fig. 2c). This suggests *SOX2* expression promotes bladder cancer cell growth.

### SOX2 promotes the survival of bladder cancer cells by activating AKT signaling

**Figure 3:**
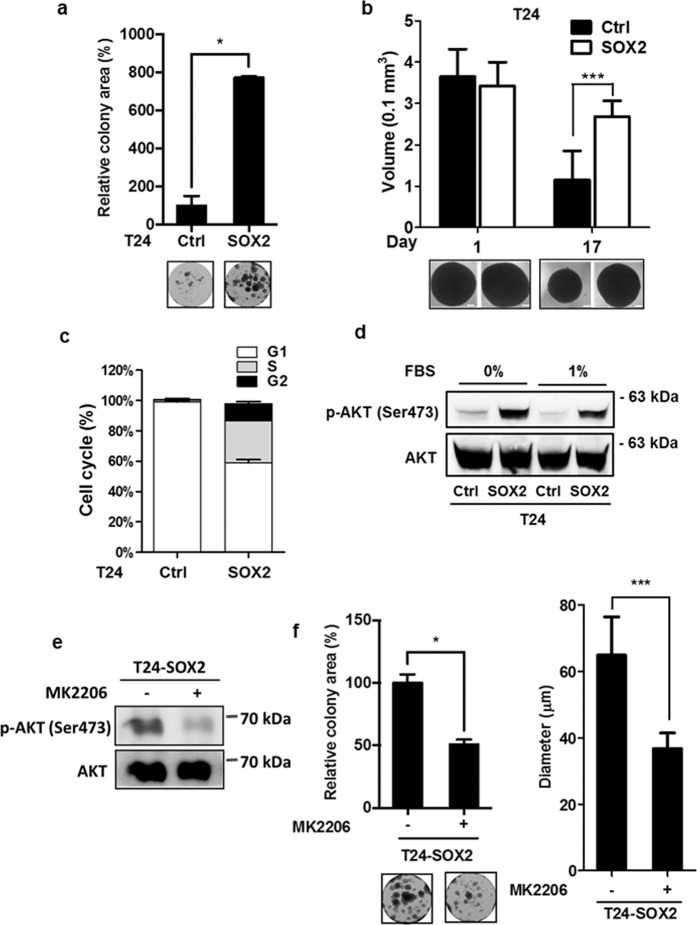
SOX2 enhances bladder cancer cell survival via activation of AKT signaling. (**a**) Clonogenic analysis to assess the serum starvation effect on T24 cells transduced with the lentiviral vector encoding *SOX2* cDNA (SOX2) or empty control vector (Ctrl) for 14 days. Colonies were cultured under low-serum (1% FBS) condition and subjected to crystal violet staining, followed by quantification of ImageJ analysis. Representative plates were photographed (bottom). Results are the average of three replicates and expressed as the mean ± S.D. *P < 0.05. (**b**) Spheroid formation analysis to assess low-serum spheroid forming ability of T24 cells transduced with the lentiviral vector encoding *SOX2* cDNA (SOX2) or empty control vector (Ctrl). *SOX2*-expressing (SOX2) or control (Ctrl) T24 cells were cultured under the low-attached culture condition in normal-serum medium (10% FBS) for 7 days and subjected to low-serum medium (1% FBS) for another 17 days. Photos are representative images of the spheroids at day 1 and day 17 under low-serum condition (bottom). Results are the average of three independent experiments and expressed as the mean ± S.D. ***P < 0.001. (**c**) Flow cytometry analysis to assess cell-cycle distribution under low-serum condition in *SOX2*-expressing (SOX2) or control (Ctrl) T24 cells. The transduced cells were cultured under low-serum (1% FBS) condition for 48 hr and subjected to cell-cycle analysis. (**d**) Immunoblotting analysis to assess the expression of phosphorylated AKT at Ser473 and total AKT in *SOX2*-expressing (SOX2) or control (Ctrl) T24 cells under serum-free or low-serum condition (1% FBS) for 48 hr. (**e**) Immunoblotting analysis to assess the expression of phosphorylated AKT at Ser473 and total AKT expression in *SOX2*-expressing T24 cells under low-serum condition (1% FBS) in the presence or absence of MK2206 (1 μM) for 48 hr. (**f**) Clonogenic assay (left) and 3D colony-forming analysis (right) of *SOX2*-expressing T24 cells under low-serum condition (1% FBS) in the presence or absence of MK2206 (1 μM) for 14 days. Colonies were quantified by ImageJ analysis. Results are the average of three independent experiments and expressed as the mean ± S.D. *P < 0.05, ***P < 0.001.

To test whether *SOX2* plays a role in cell survival, we assessed *SOX2* expression in T24 cells under a low-serum stress. Clonogenic analysis showed that *SOX2* expression promoted T24 cell growth under a low-serum (1% FBS) condition (Fig. 3a). We further validated the effect of *SOX2* expression on T24 cell-spheroid formation under low-serum stress. The T24 cells formed spheroids in a 3D culture system under the normal-serum (10% FBS) condition, wherein *SOX2* expression did not affect spheroid formation (Fig. 3b). By contrast, long-term culturing of T24 spheroids under low-serum condition (1% FBS) attenuated the size of the spheroids; however, *SOX2* expression sustained the T24 spheroid-forming capability under the low-serum condition, indicating *SOX2* is involved in bladder cancer cell survival (Fig. 3b). In addition, the cell cycle analysis revealed that *SOX2* expression sustained the S-phase in T24 cells under the low-serum condition (Fig. 3c and Supplementary Figure S2c bottom left). These findings suggest that *SOX2* expression helps bladder cancer cells overcome low-serum stress.

Because AKT signaling contributes to cell survival, we examined *SOX2*’s effect on AKT (Ser473) phosphorylation. Immunoblotting analysis revealed that SOX2 expression in T24 cells sustained AKT phosphorylation under serum-free and low-serum (1% FBS) conditions (Fig. 3d). Moreover, knockdown of SOX2 decreased AKT phosphorylation in 5637 cells (Supplementary Figure S3a). To further verify whether AKT activation mediates *SOX2*-induced bladder cancer survival, we pharmacologically inhibited AKT activation with MK2206. MK2206 treatment reduced the AKT phosphorylation in 5637 cells as well as in *SOX2*-expressing T24 cells (Fig. 3e, Supplementary Figure S3b). Clonogenic and 3D colony-forming assays revealed MK2206 also reduced the colony numbers and sizes with respect to the *SOX2*-expressing T24 cells (Fig. 3f). These results highlight *SOX2*’s activation of AKT signaling to boost bladder cancer cell survival.

### IGF2 expression is regulated by SOX2 under an epigenetic control

**Figure 4:**
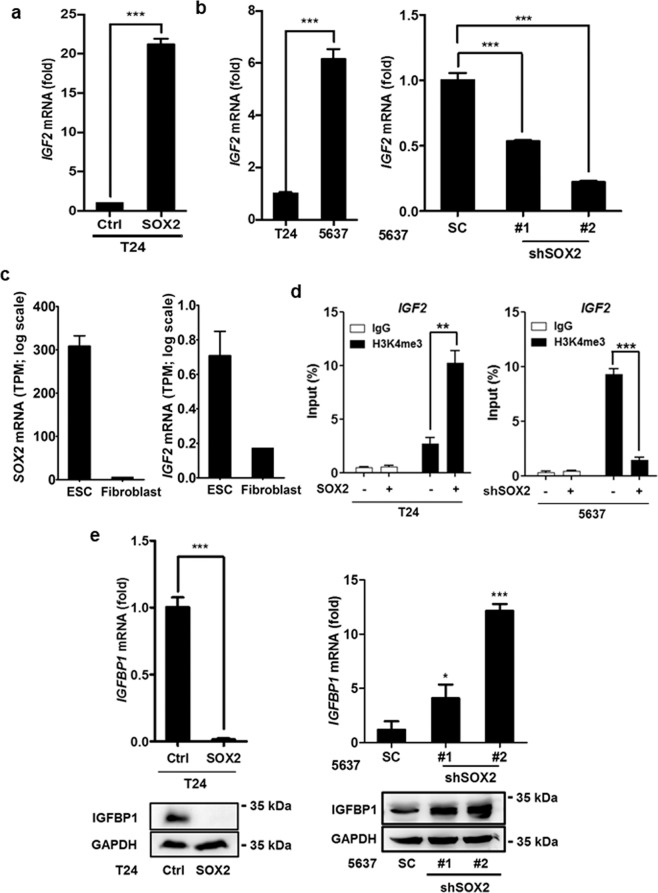
SOX2 induces IGF2/IGF1R signaling molecules in bladder cancer cells. (**a**) qPCR analysis to assess *IGF2* expression in T24 cells transduced with the lentiviral vector encoding *SOX2* cDNA (SOX2) or empty control vector (Ctrl). Results are the average of three replicates and expressed as the mean ± S.D. ***P < 0.001. (**b**) qPCR analysis to assess *IGF2* expression in T24 versus 5637 cells (left) and in 5637 cells transduced with shSOX2 versus scrambled control (SC) vector (right). The #1 and #2 indicate the two distinct shRNAs that target different regions within *SOX2*. ***P < 0.001. (**c**) RNA-seq analysis of *SOX2* and *IGF2* expression in ESC (HUES3) and differentiated fibroblast cells from GSE73211 database. (**d**) ChIP-qPCR analysis to assess H3K4me3 levels of *IGF2* region in SOX2-expressing T24 cell (left) and SOX2-silenced 5637 cells (right). **P < 0.01, ***P < 0.001. Primers targeting the H3K4me3 signal of *IGF2* were designed at chr11: 2,158,962–2,159,038. (**e**) qPCR and immunoblotting analysis of *IGFBP1* expression in SOX2-overexpressing T24 cells (left) and SOX2-silenced 5637 cells (right). Results are the average of three replicates and expressed as the mean ± S.D. *P < 0.05, ***P < 0.001.

To identify the genes responsible for *SOX2*-mediated bladder cancer cell survival, gene expression profiling analysis was performed using the *SOX2*-expressing T24 cells and control T24 cells, and the result was uploaded as GSE145826. qPCR analysis confirmed *IGF2* levels were considerably higher in *SOX2*-expressing T24 than in control T24 cells (Fig. 4a). Similarly, SOX2-high 5637 cells exhibited higher *IGF2* level than SOX2-low T24 cells, while *SOX2* silencing in 5637 cells inhibited *IGF2* expression (Fig. 4b). Because SOX2 and IGF2 expression has been linked to embryonic development, we examined *SOX2* and *IGF2* expression in ESC and fibroblasts. RNA-seq and qPCR assays showed that both *SOX2* and *IGF2* levels were downregulated in the differentiated fibroblasts compared to ESC (Fig. 4c and Supplementary Figure S4). Since SOX2 mediates stem cell differentiation and iPSC reprogramming mainly via epigenetic regulation, whereby H3K4me3 marks sites with active gene expression^35,36^, we examined whether H3K4me3 signal on the *IGF2* locus is affected by SOX2 expression in bladder cancer cells. ChIP-seq analysis showed a strong H3K4me3 signal on the *IGF2* locus in ESC but not in fibroblasts (Supplementary Figure S5). ChIP-qPCR analysis revealed the H3K4me3 signal on the *IGF2* locus increased because of *SOX2* expression in T24 cells and decreased on account of SOX2 knockdown in 5637 cells (Fig. 4d). We also found *IGFBP1* expression decreased because of *SOX2* expression in T24 cells and increased on account of SOX2 knockdown in 5637 cells (Fig. 4e). These results indicate *SOX2* expression regulates *IGF2* signaling.

### *IGF2/IGF1R* signaling is essential for SOX2-mediated bladder cancer cell survival

**Figure 5:**
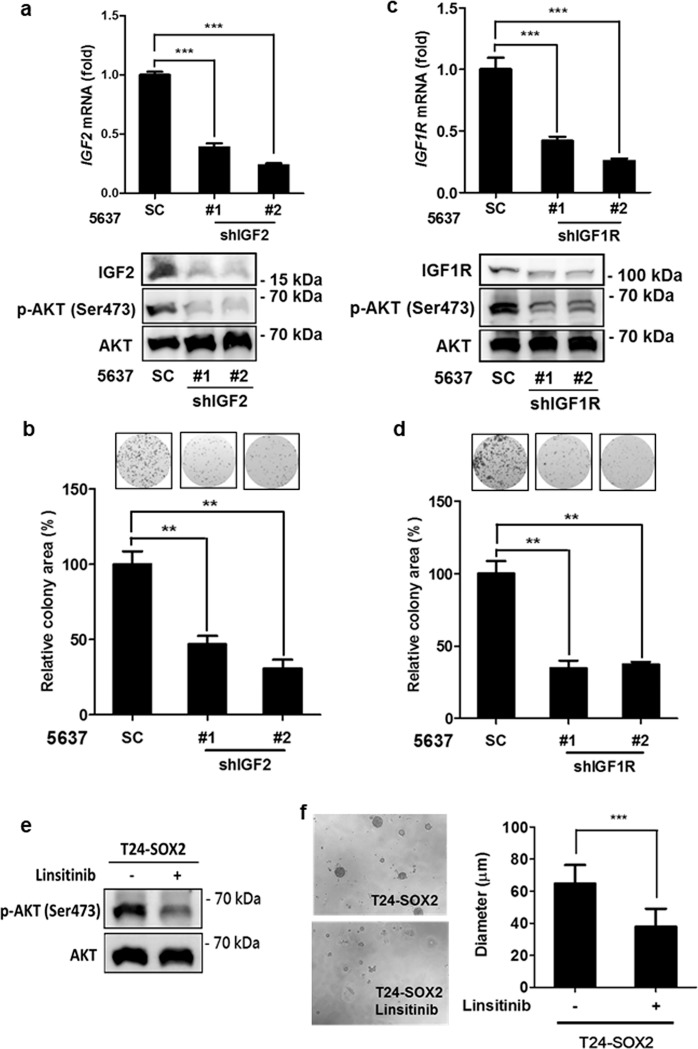
SOX2-induced *IGF2/IGF1R* signaling is essential for bladder cancer cells. (**a**) qPCR (upper) and immunoblotting (lower) analysis of 5637 cells transduced with the lentiviral vector encoding shRNA against *IGF2* (shIGF2) or scrambled control vector (SC). The #1 and #2 indicate the two distinct shRNAs that target different regions within *IGF2*. ***P < 0.001. (**b**) Clonogenic assay of 5637 cells transduced with shIGF2 or scrambled control vector (SC). Colonies were subjected to crystal violet staining (top) and quantified by ImageJ analysis. Results are the average of three replicates and expressed as the mean ± S.D. **P < 0.01. (**c**) qPCR (upper) and immunoblotting (lower) analysis of 5637 cells transduced with the lentiviral vector encoding shRNA against *IGF1R* (shIGF1R) or scrambled control vector (SC). The #1 and #2 indicate the two distinct shRNAs that target different regions within *IGF1R*. ***P < 0.001. (**d**) Clonogenic assay of 5637 cells transduced with shIGF1R or scrambled control vector (SC). Colonies were subjected to crystal violet staining (top) and quantified by ImageJ analysis. Results are the average of three replicates and expressed as the mean ± S.D. **P < 0.01. (**e**) Immunoblotting analysis to assess the expression of phosphorylated AKT at Ser473 and total AKT expression in *SOX2*-expressing T24 cells under low-serum (1% FBS) condition in the presence or absence of linsitinib (5 μM) for 48 hr. (**f**) 3D colony-forming analysis to assess linsitinib effect on colony formation of *SOX2*-expressing T24. The cells were grown under the low-attached condition in low-serum medium (1% FBS) in the presence or absence of linsitinib (5 μM) for 7 days. Photos (left) are representative images of the colony and colony sizes were quantified (right). Results are the average of three independent experiments and expressed as the mean ± SD. ***P < 0.001.

To understand whether *IGF2/IGF1R* signaling is responsible for high-*SOX2* expressing bladder cancer cell growth, we knocked down *IGF2* and *IGF1R* (Fig. 5a–d). Clonogenic assays showed that *IGF2* and *IGF1R* knockdown weakened the colony-forming capability of the high-SOX2 expressing 5637 cells (Fig. 5b,d). Immunoblotting assays revealed that AKT phosphorylation decreased on account of knockdown of *IGF2* or *IGF1R* in 5637 cells (Fig. 5a,c). Linsitinib, an IGF1R inhibitor, was used to block *IGF2/IGF1R* signaling-mediated AKT phosphorylation in *SOX2*-expressing bladder cancer cells. Immunoblotting assays revealed that linsitinib inhibited AKT phosphorylation in 5637 cells as well as in *SOX2*-expressing T24 cells (Fig. 5e and Supplementary Figure S3b). 3D colony-forming assays revealed that linsitinib reduced colony formation of *SOX2*-expressing T24 cells (Fig. 5f). This suggests that *IGF2* and *IGF1R* are crucial for SOX2-mediated growth and survival of bladder cancer cells.

### *IGF1R* signaling serves as a prognostic biomarker for bladder cancer

**Figure 6:**
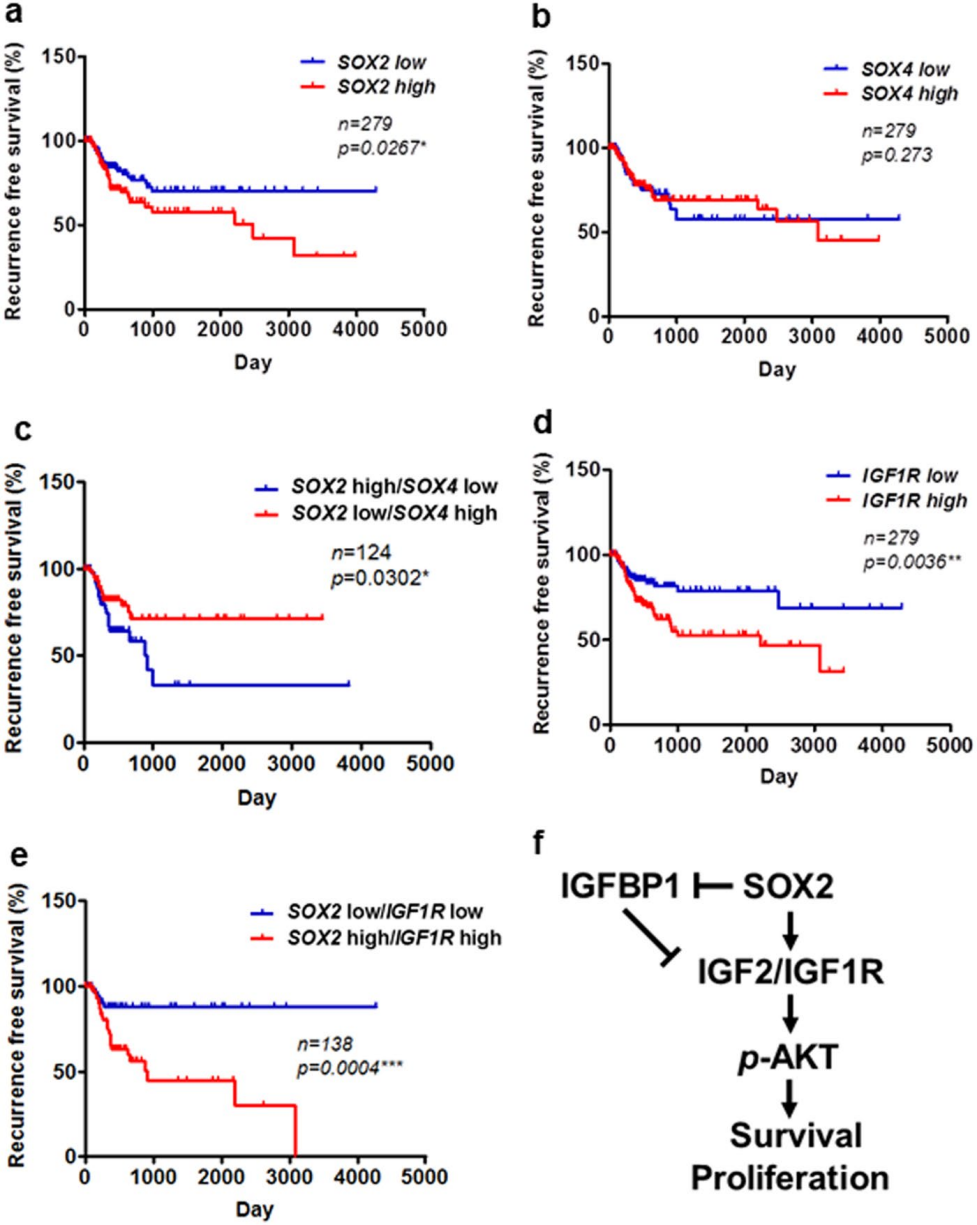
*SOX2* and *IGF1R* as prognostic factors in bladder cancer. (**a,b**) Kaplan–Meier analysis of *SOX2* (**a**) and *SOX4* (**b**) expression with recurrence-free survival in patients from TCGA_BLCA cohort. The significance was examined by log-rank test. *P < 0.05. (**c**) Kaplan–Meier analysis to assess the correlation of *SOX2-*high/*SOX4-*low and *SOX2-*low/*SOX4-*high signatures with recurrence-free survival in patients from TCGA_BLCA cohort. The significance was examined by log-rank test. *P < 0.05. (**d**) Kaplan–Meier analysis of *IGF1R* expression with recurrence-free survival in patients from TCGA_BLCA cohort. The significance was examined by log-rank test. **P < 0.01. (**e**) Kaplan–Meier analysis to assess the correlation of *SOX2-*high/*IGF1R-*high and *SOX2-*low/*IGF1R*-low signatures with recurrence-free survival in patients from TCGA_BLCA cohort. The significance was examined by log-rank test. ***P < 0.001. (**f**) The schematic model of SOX2-mediated oncogenesis of bladder cancer. SOX2 induces the expression of *IGF2* and *IGF1R*, but suppresses that of *IGFBP1*, thus promoting AKT phosphorylation, with increased proliferation and survival of bladder cancer cells.

Our present results confirmed that *SOX2* regulates *IGF2*/*IGF1R* signaling in bladder cancer cells. *SOX4*, another *SOX* family member, is reported to be amplified in bladder cancer^14,15^. We confirmed that *SOX4*, but not *SOX2*, was mainly amplified in primary bladder tumors (Supplementary Figure S6). To further examine the potential of *SOX2*, *SOX4*, and *IGF1R* signaling in bladder cancer prognosis, we correlated the expression of these molecules with recurrence-free survival in primary bladder tumors. Kaplan-Meier analysis showed that *SOX2*, but not *SOX4*, expression correlated with a poor recurrence-free survival in bladder cancer patients, and the patients harboring a *SOX2*-high/*SOX4*-low signature had a worse recurrence-free survival outcome than those with *SOX2*-low/*SOX4*-high signature (Fig. 6a–c). Moreover, *SOX4*, but not *SOX2*, correlates with a good overall survival, and patients harboring the *SOX4*-low/*SOX2*-high signature show a worse overall survival than those with the *SOX4*-high/*SOX2*-low signature (Supplementary Figure S7). Furthermore, *IGF1R* expression was also associated with a poor recurrence-free survival outcome (Fig. 6d). We observed that the patients harboring a *SOX2*-high/*IGF1R*-high signature had a worse recurrence-free survival outcome than those with *SOX2*-low/*IGF1R*-low signature (Fig. 6e). Both univariate and multivariate regression assays of IGF signaling molecules showed that *IGF1R* is the only independent predictor of poor recurrence-free survival (Table 1). These findings suggest that *IGF1R* can be a potential biomarker for predicting poor survival outcomes.

**Table 1:** Univariate and multivariate analyses of IGF signaling factors on recurrence-free survival in bladder cancer patients from TCGA_BLCA cohort.

Variable			Univariate analysis		Multivariate analysis	
			Hazard ratio (95% CI)	P value	Hazard ratio (95% CI)	P value
*IGF1*	<	Mean	1	0.1807	1	0.1715
	≥	Mean	1.390 (0.858–2.250)		1.417 (0.860–2.336)	
*IGF2*	<	Mean	1	0.7112	1	0.4848
	≥	Mean	0.911 (0.557–1.490)		0.832 (0.497–1.393)	
*IGF1R*	<	Mean	1	0.0066**	1	0.0062**
	≥	Mean	2.093 (1.228–3.568)		2.136 (1.241–3.677)	
*IGFBP1*	<	Mean	1	0.5761	1	0.5313
	≥	Mean	1.151 (0.702–1.888)		1.188 (0.693–2.037)	
*IGFBP3*	<	Mean	1	0.6081	1	0.7504
	≥	Mean	0.881 (0.543–1.430)		0.915 (0.531–1.578)	
*IGF2BP3*	<	Mean	1	0.8349	1	0.5824
	≥	Mean	1.053 (0.650–1.705)		0.867 (0.522–1.441)	

## Discussion

Although signaling elicited by the stem cell factors SOX2, OCT4, KLF4, and MYC has been associated with cancer progression in several tumors, it has remained unclear how these signaling molecules mediate bladder cancer progression. We found that *SOX2*, but not *OCT4*, *KLF4*, or *MYC* expression, correlates with poor prognosis and histologic differentiation in bladder cancer. Moreover, we found that *SOX2* promotes bladder cancer cell survival by inducing the *IGF2/IGF1R* pathway, thereby activating AKT survival signaling. Pharmacological inhibition of IGF1R or AKT inhibits bladder cancer cell survival. Our findings provide insights on SOX2-mediated oncogenesis in bladder cancer and highlight putative therapeutic targets.

Because the introduction of *SOX2*, *OCT4*, *MYC*, and *KLF4* is sufficient to reprogram differentiated cells into iPSC with ESC properties, the combined expression of these factors has been hypothesized to initiate tumors and promote cancer progression^37–40^. On the one hand, tumors harboring ESC-like gene expression signature with active targets of *SOX2, OCT4, and MYC* were associated with poor pathological differentiation and poor prognosis in brain, breast, and bladder cancer patients^6^. On the other hand, tumor malignancy has been attributed only to *MYC* and not to other ESC factors^41^. Here, we observed that only *SOX2* expression correlates with poor recurrence-free survival of bladder cancer patients. Moreover, we found that *SOX2* expression is associated with poor pathological differentiation, emphasizing its involvement in bladder cancer malignancy. This is in accordance with the results of Ruan *et al*., who reported that SOX2 and Ki67 expression in T1 early stage, non-muscle, invasive bladder tumors correlate with poor recurrence-free survival^18^. Currently, SOX2 is thought to be a cancer stem cell marker in relation to bladder tumors and Sox2 knockout within primary invasive bladder cancer caused enhanced tumor regression^42^. *SOX4*, another *SOX* family member, is overexpressed in bladder tumors harboring the genomic 6p22 amplification^15,43,44^. Here, we observed that *SOX4* is mainly amplified in primary bladder tumors, whereas only *SOX2* expression is associated with poor recurrence-free survival in patients with bladder cancer. *SOX2* expression did not affect *SOX4* levels in T24 cells (Supplementary Figure S8). Together, these data suggest that *SOX4* and *SOX2* play distinct roles in tumor initiation and progression.

The molecular mechanism linking *SOX2* expression to poor prognosis in bladder cancer has not been well understood. We found that *SOX2* expression not only promotes cell proliferation but also enhances cell survival under low-serum stress. We discovered that, under low-serum stress, *SOX2* expression induces AKT phosphorylation and sustains bladder cancer cells’ spheroid-forming capability. In many cancers, AKT phosphorylation promotes cell survival by inducing drug resistance and desensitizing radiation therapy. We observed that pharmacological inhibition of AKT phosphorylation attenuates bladder cancer cells’ *SOX2*-mediated survival and spheroid-forming capability. Furthermore, we identified IGF2 and IGF1R as AKT upstream molecules which induce AKT phosphorylation in *SOX2*-positive bladder cancer cells. *IGF2* expression boosts cancer cell survival and tumor progression in colon cancer^45,46^. To search for *SOX2*-binding targets in colon cancer cells, Fang *et al*. performed ChIP-seq analysis and identified *IGF1R* as one of direct binding targets of *SOX2*^47^. We observed that *SOX2* expression in T24 cells increased *IGF1R* expression, whereas *SOX2* knockdown in 5637 cells did not affect *IGF1R* expression (Supplementary Figure S9). In contrast, *SOX2* expression increased *IGF2* expression, and *SOX2* knockdown decreased *IGF2* level, supporting *IGF2* as a potential downstream target of SOX2. Our ChIP-qPCR analysis did not show *SOX2* binding to the *IGF2* locus (data not shown). In addition to directly binding to target genes, *SOX2* regulates gene expression via histone modifications during stem cell differentiation and lung cancer cell plasticity^16,48^. ChIP-qPCR analysis showed that the signal of H3K4me3, a predominant mark of active promoters, increased upon *SOX2* expression in bladder cancer cells. Knockdown of *IGF2* and *IGF1R* attenuated the growth of bladder cancer cells. All these data verify *SOX2* promotes bladder cancer cell growth and survival by inducing *IGF2/IGF1R* signaling.

Currently, limited targeted therapies are available for treating this aggressive disease. Since our evidence suggests that *SOX2* activates AKT survival signaling and promotes spheroid-forming ability by inducing *IGF2/IGF1R* in bladder cancer cells, IGF2 and IGF1R may be potential therapeutic targets for treating bladder cancer. IGF2 expression promotes aggressiveness in lethal prostate cancer and has been proposed to be a druggable target^31^. Indeed, we found that pharmacological inhibition of IGF2/IGF1R signaling with linsitinib decreased AKT phosphorylation and attenuated bladder cancer cells’ *SOX2*-mediated colony-forming ability. These findings highlight IGF2/IGF1R’s potential as therapeutic targets to treat bladder cancer. Moreover, we observed that *IGF1R* expression correlates with poor recurrence-free survival in bladder cancer patients. The tumors harboring high-*SOX2*/high-*IGF1R* signature are associated with the worst survival outcome in bladder cancer patients. These data suggest *IGF1R* may serve as a novel biomarker for monitoring bladder cancer progression.

The limitation of this study is that we mainly used T24 and 5637 cells, which represent muscle invasive bladder cancer cell lines maintained in different media, to dissect the potential oncogenic role of SOX2 in bladder cancer. Thus, these data may not be sufficient to represent all bladder cancer. Through gene expression analysis, 5637 cells have been classified into basal subtype bladder cancer, which displays cancer stem cell markers and is associated with poor prognosis^49–51^. SOX2 has been characterized as s a marker for stem-like tumor cells in bladder cancer^42^. Hence, our findings that SOX2 activates IGF2/IGF1R signaling and predicts poor prognosis further link IGF2/IGF1R signaling to SOX2 mediated aggressiveness in bladder cancer.

In conclusion, we demonstrated that *SOX2* stimulated *IGF2* expression to activate AKT signaling, enhancing the survival and spheroid-forming capability of bladder cancer cells. Moreover, we observed *SOX2* and *IGF1R* levels are significantly correlated with poor prognosis in bladder cancer patients. The fact that *SOX2–IGF2/IGF1R* signaling axis confers aggressiveness in bladder cancer suggests that *IGF2/IGF1R* may serve as therapeutic targets in treating bladder cancer.

## Materials and Methods

### Chemicals and reagents

Linsitinib (OSI-906) was purchased from Cayman Chem (Ann Arbor, MI, USA). MK2206 was ordered from LC Laboratories (Woburn, MA, USA).

### Cell culture

T24 and 5637 cell lines were obtained in 2017 from BCRC (Bioresource Collection and Research Center, Taiwan). T24 and 5637 cells were cultured in Dulbecco’s modified Eagle’s medium (DMEM) and RPMI-1640, respectively, supplemented with 10% fetal bovine serum (FBS).

### RNA extraction and qPCR

Total RNA was extracted from cells using REzol reagent (Protech Technology Enterprise CO., Ltd., Taiwan) according to the manufacturer’s protocol. Quantitative real-time PCR (qPCR) was conducted with the StepOneTM Real-Time PCR system (Applied Biosystems; ABI, USA)^52^. Amplification of specific genes was detected using the UPL universal probe system (Roche Life Science, USA) or specific synthesized probes (Integrated Device Technology; IDT, USA). The data were normalized to the housekeeping gene 18 S rRNA. For the detailed RT-primer and probe sequences, see Supplementary Table S2.

### Plasmids

SOX2 expressing vector was prepared as described previously^11^. pLKO.1-shSOX2 #1 (TRCN0000003252), pLKO.1-shSOX2 #2 (TRCN0000010772), pLKO.1-shIGF2 #1 (TRCN0000062430), pLKO.1-shIGF2 #2 (TRCN0000062432), pLKO.1-shIGF1R #1 (TRCN0000121193) and pLKO.1-shIGF1R #2 (TRCN0000121301) were obtained from the National RNAi Core Facility, Academia Sinica (Taipei, Taiwan). pLKO.1-Scrambled shRNA was obtained from Addgene. Lentiviral production and infection were performed using the previously described method^53^.

### Trypan blue cell exclusion assay

Cells (10^5^ cells/well) were seeded in 24-well plates and incubated in triplicate tests. In indicated times, cells were trypsinized and then counted using Trypan Blue staining in low magnification under the microscope.

### Cell-cycle analysis by flow cytometry

Cells were plated at a density such that they would be 50% confluent on the day of analysis. Cells were trypsinized and fixed in cold 70% ethanol for 10 min and then stained with propidium iodide solution (1 mM) at room temperature for 30 min. The stained cells were analyzed using BD Accuri C6 flow cytometer with excitation at 488 nm and emission at 617 nm.

### AlamarBlue cell proliferation assay

To perform the cell proliferation assay, cells were seeded at 5 × 10^2^ cells per well in 96-well plate. The cell growth at indicated time interval was monitored using alamarBlue (Thermo Scientific) according to manufacturer’s instructions. Cell titers were calculated by measuring the absorbance at 585 nm by VICTOR2 D fluorometer (PerkinElmer).

### Clonogenic assay

Cells were plated in 24-well plates with a density of 100 cells/well. Cells were incubated for 2 weeks, and the surviving colonies were fixed and stained with crystal violet. Colonies were quantified by ImageJ software.

### Spheroid assay

24-well cell culture plates were coated with 0.7% agarose, and 1 × 10^5^ cells/well were seeded. The seeded cells were then placed on an orbital shaker with shaking at 60 rpm for 7 days to form one spheroid/well. Spheroids were then subjected to low serum (1% FBS) treatment for the indicated time periods. Spheroid volumes were calculated by using the formula V = (4/3)*pi*r^3^, where the radius r was measured by the microscope.

### 3D Colony-forming assay

60-mm plastic tissue-culture petri plates were coated with 0.7% agarose, and 200 cells/plate were seeded. The seeded cells were then placed into the incubator and incubated for 7 days to form unattached floating spheroid colonies. The floating spheroid colonies were then subjected to the indicated treatment for another 7 days. The volumes of spheroid colonies were calculated by using the formula V = (4/3)*pi*r^3^, where the radius r was measured by the microscope.

### Immunoblotting

Cells were harvested in RIPA lysis buffer supplemented with a protease inhibitor cocktail, followed by immunoblotting with SOX2 antibody (1:1000 dilution, GTX101506, GeneTex), p-AKT (Ser473, 1:1000 dilution, GTX28932, GeneTex), AKT (1:1000 dilution, #1085-1, EPITOMICS), IGF2 (1:1000 dilution, GTX129110, GeneTex), IGF1R (1:1000 dilution, GTX50433, GeneTex), IGFBP1 (1:1000 dilution, GTX129006, GeneTex), and GAPDH (1:10,000 dilution, GTX100118, GeneTex).

### Immunohistochemistry (IHC) staining

IHC was conducted as previously described^16^. The primary SOX2 antibody used in the IHC was SOX2 antibody (1:400 dilution, PM056, MBL). The immunoreactivity pattern and histologic appearance of the tissue array (BLC661, US BIOMAX) were examined and scored by the pathologist (Supplementary Table S3). The Allred scoring system was used to give the staining scores for the expression of SOX2 based on the sum of the percentage of cells that were stained by IHC (on a scale of 0–5) and the intensity of the staining (on a scale of 0–3). The final score was determined by the sum of the intensity scores and the positivity scores of stained cells.

### ChIP-qPCR

ChIP-qPCR was performed using the protocol as previously described^16^, with the antibodies against H3K4me3 (GTX128954, GeneTex) or control IgG (GTX35035, GeneTex). For the detailed RT-primer and probe sequences used in ChIP-qPCR, see Supplementary Table S2.

### Public domain data analysis

Public gene expression profiling datasets of bladder cancer patients were accessed from TCGA_BLCA database via UCSC cancer browser, and GSE32894 and GSE73211 via GEO browser. The means of *SOX2*, *SOX4*, *KLF4*, *MYC*, *OCT4*, and *IGF1*R expression were used as cut-off points for statistics analysis. The ChIP-seq results of H3K4me3 epigenetic regulation in embryonic stem cells and fibroblasts were collected from ENCODE (https://www.encodeproject.org/). The genetic alteration rates of *SOX2* and *SOX4* in bladder cancer were analyzed from cBioPortal (http://www.cbioportal.org/). For the used public domain databases, see Supplementary Table S4.

### Statistical analysis

The attribution of different gene expression and clinical associated variables in death risks of bladder cancer were calculated using the Cox’s proportional hazards regression analysis and demonstrated in forest plot or tables. Recurrence-free survival and overall survival curves of bladder cancer patients were analyzed by the Kaplan-Meier plots and compared the difference between groups by the log-rank test. All statistics analysis was presented using SAS software, version 9.4. Significance difference was set in which P value was less than 0.05.

## Supplementary information

Supplementary Information.
