# Supplementary material for: Critical role of SOX2–IGF2 signaling in aggressiveness of bladder cancer
Source: Sci Rep. 2020 May 19;10:8261. doi: 10.1038/s41598-020-65006-z (PMC7237425; doi:10.1038/s41598-020-65006-z)
Supplement: Supplementary file 1 — Supplementary Information. [file 41598_2020_65006_MOESM1_ESM.pdf]

Supplementary Information

Critical role of SOX2–IGF2 signaling in aggressiveness of bladder cancer

Yu-Fan Chiu, Chia-Chang Wu, Ming-Han Kuo, Chia-Cheng Miao, Ming-Yi Zheng, Pei-Yu Chen, Sheng-Chieh Lin, Junn-Liang Chang, Yuan-Hung Wang, Yu-Ting Chou

.

Supplementary information includes

Table S1. Univariate and multivariate analyses of clinical parameters and *SOX2*, *KLF4*, *MYC* and *OCT4* stem cell factors on recurrence-free survival in bladder cancer patients from TCGA_BLCA cohort.
Figure S1. *SOX2* expression in bladder cancer cells.
Figure S2. SOX2 regulates cell cycle progression in bladder cancer cells.
Figure S3. SOX2 regulates AKT phosphorylation.
Figure S4. Differential expression of *SOX2* and *IGF2* in embryonic stem cells and fibroblasts.
Figure S5. Differential H3K4me3 modification of IGF2 in ESC and fibroblast.
Figure S6. Gene copy-number variation analysis of *SOX2* and *SOX4* in bladder tumors.
Figure S7. *SOX4* signifies a good survival in bladder cancer patients.
Figure S8. *SOX4* expression is not affected by *SOX2* expression in bladder cancer cells.
Figure S9. Effect of *SOX2* expression on *IGF1R* expression.
Table S2. Primer and probe sequence list.
Table S3. The clinicopathologic information of bladder cancer tissues from BLC661 tissue array (US BIOMAX).
Table S4. Public domain data used in this study.
Table S5. Spheroid analysis of three independent assays
Table S6. 3D colony-forming analysis of three independent assays
Figure S10-S17. The images of full-length blots of western analysis

Table S1. Univariate and multivariate analyses of clinical parameters and stem cell factors *SOX2*, *KLF4*, *MYC* and *OCT4* on recurrence-free survival in bladder cancer patients from TCGA_BLCA cohort

Variable		Univariate analysis		Multivariate analysis	
		Hazard ratio (95% CI)	P value	Hazard ratio (95% CI)	P value
Gender	Female	1		1	
	Male	0.577 (0.293-1.137)	0.1118	0.629 (0.307-1.292)	0.2072
Stage	I、II	1		1	
	III、IV	2.749 (1.152-6.561)	0.0227*	0.687 (0.108-4.356)	0.6901
T	T0、T1、T2	1		1	
	T3	3.132 (1.297-7.562)	0.0112*	3.196 (0.541-18.871)	0.1997
	T4	2.716 (0.865-8.531)	0.087	1.501 (0.209-10.776)	0.6864
N	N0	1		1	
	N1、N2、N3	2.41 (1.29-4.504)	0.0058**	1.833 (0.873-3.847)	0.1093
M	M0	1		1	
	M1	3.958 (0.927-16.894)	0.0632	1.978 (0.383-10.231)	0.4158
SOX2	< Mean	1		1	
	≥ Mean	2.467 (1.292-4.709)	0.0062**	2.966 (1.451-6.064)	0.0029**
KLF4	< Mean	1		1	
	≥ Mean	1.185 (0.636-2.206)	0.5933	1.233 (0.595-2.557)	0.5734
MYC	< Mean	1		1	
	≥ Mean	1.807 (0.942-3.463)	0.0748	1.767 (0.841-3.714)	0.1332
OCT4	< Mean	1		1	
	≥ Mean	0.373 (0.192-0.724)	0.0035**	0.432(0.215-0.869)	0.0186*

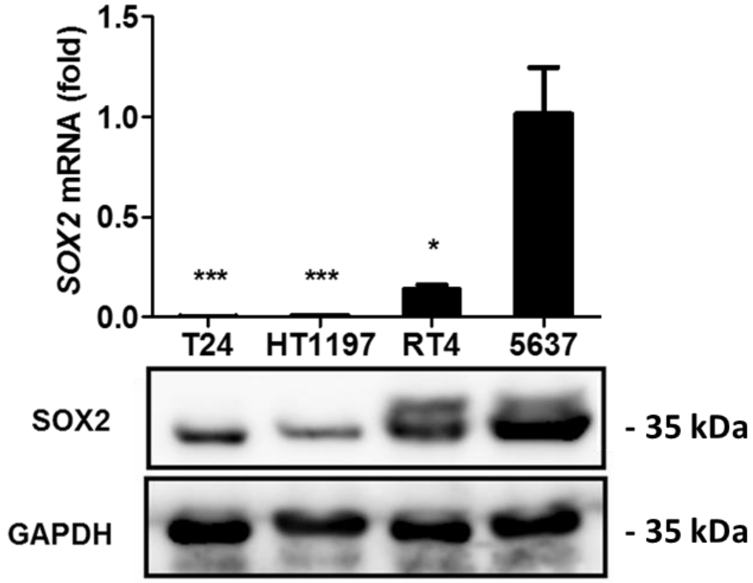

Figure S1. ***SOX2*** **expression in bladder cancer cells.**

qPCR (upper) and immunoblotting (lower) analysis to assess *SOX2* expression in bladder cancer cell line T24, HT1197, RT4, and 5637.

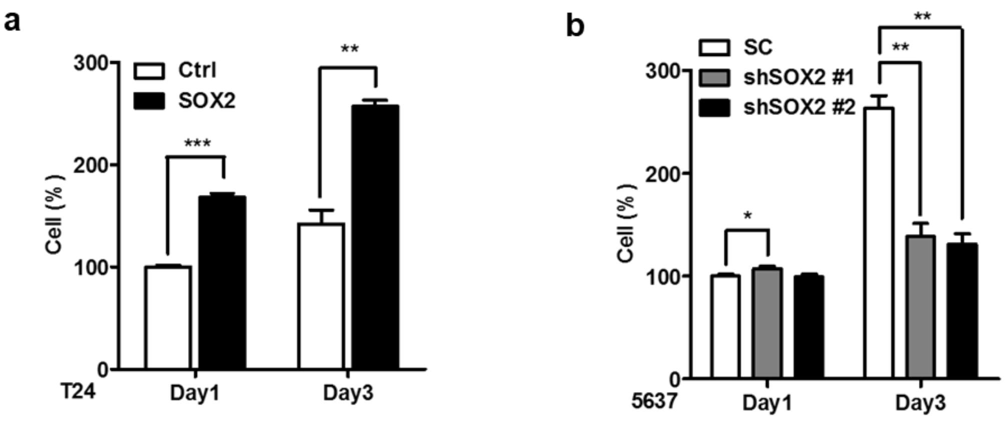

c

5637	G1	S	G2/M
SC	54.9%	46.2%	1.3%
shSOX2 #1	60.2%	39.1%	3.2%
shSOX2 #2	59.7%	37.9%	2.1%

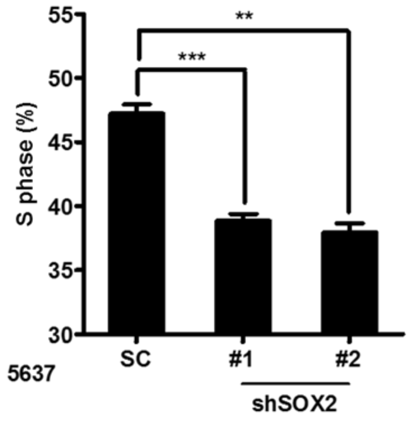

Figure S2. SOX2 regulates cell cycle progression in bladder cancer cells.

(a) AlamarBlue proliferation analysis (right) of T24 cells transduced with the lentiviral vector encoding SOX2 cDNA (SOX2) or empty control vector (Ctrl) for the indicated days. Results are the average of three replicates and expressed as the mean ± S.D. $^{**}P < 0.01$, $^{***}P < 0.001$. (b) AlamarBlue proliferation analysis (right) of 5637 cells transduced with the lentiviral vector encoding shSOX2 or scrambled control vector (SC) for the indicated days. Results are the average of three replicates and expressed as the mean ± S.D. $^{**}P < 0.01$. The #1 and #2 indicate the two distinct shRNAs that target different regions within *SOX2*. (c) Flow cytometry analysis to assess cell-cycle distribution of all phases (left) and S phase (right) in 5637 cells transduced with the lentiviral vector encoding shRNA against *SOX2* (shSOX2) or scrambled control vector (SC). $^{**}P < 0.01$, $^{***}P < 0.001$. The #1 and #2 indicate the two distinct shRNAs that target different regions within *SOX2*.

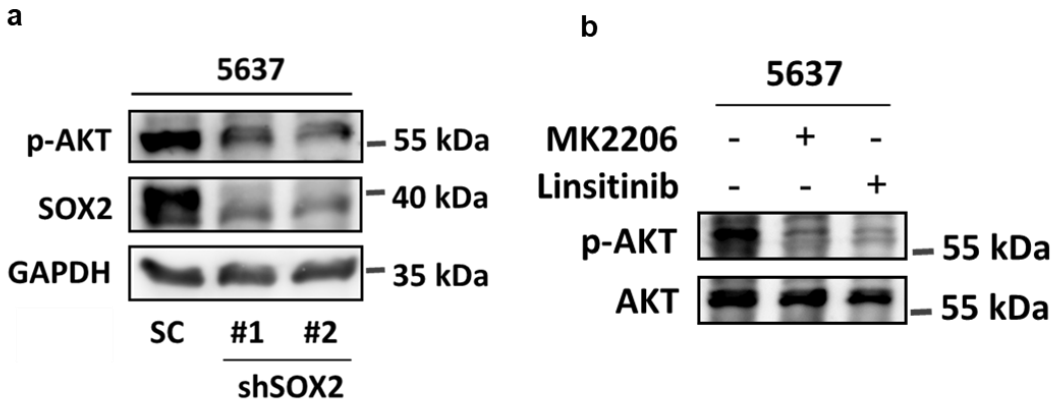

Figure S3. SOX2 regulates AKT phosphorylation.

(a) Immunoblotting analysis to assess the expression of phosphorylated AKT at Ser473 and SOX2 in 5637 cells transduced with shSOX2 or scrambled control (SC) vector (right). The #1 and #2 indicate the two distinct shRNAs that target different regions within *SOX2*. (b) Immunoblotting analysis to assess the expression of phosphorylated AKT at Ser473 and total AKT in 5637 cells treated with MK2006 (1 μM) or linsitinib (5 μM) for 48 hr.

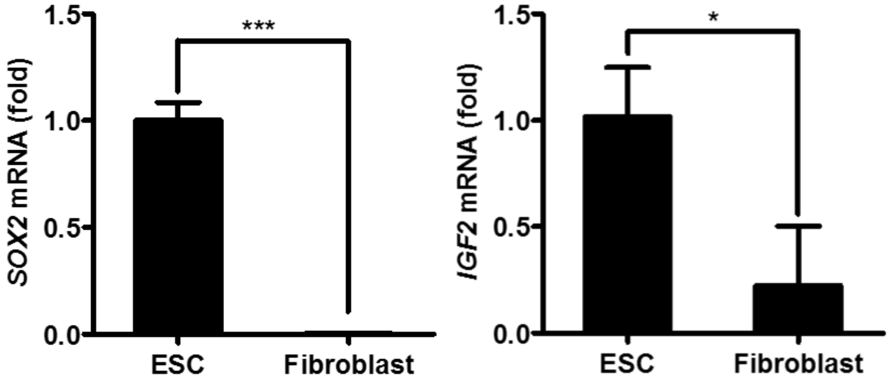

Figure S4. Differential expression of *SOX2* and *IGF2* in embryonic stem cells and fibroblasts.

qPCR analysis to assess *SOX2* (left) and *IGF2* (right) expression in ESC (H9) and derived fibroblast cells. $*P < 0.05$, $***P < 0.001$.

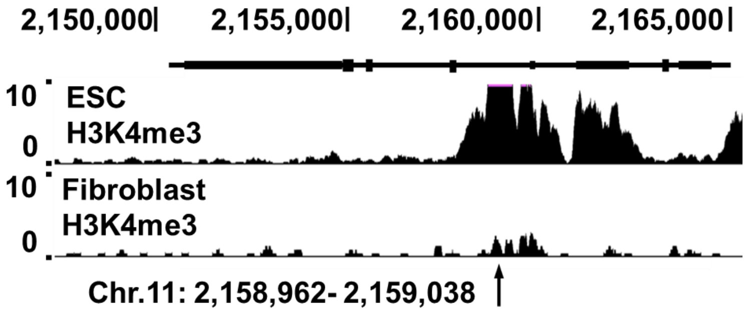

Figure S5. Differential H3K4me3 modification of *IGF2* in ESC and fibroblast. H3K4me3 ChIP-seq analysis of *IGF2* region in ESC (H1) and fibroblast (lung fibroblast) cells from ENCODE database. The arrow marks the region examined by ChIP-qPCR.

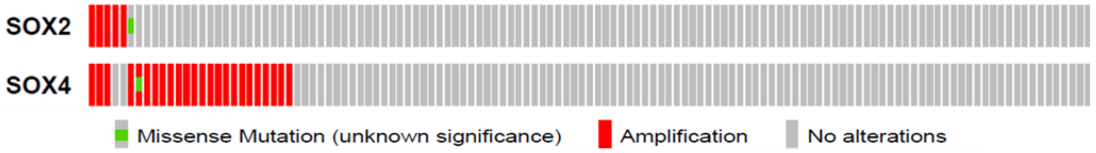

Genetic Alternation of Bladder Cancer

Gene	Amplification Frequency
SOX2	3.94% (5/127)
SOX4	18.11% (23/127)

Figure S6. Gene copy-number variation analysis of *SOX2* and *SOX4* in bladder tumors.

Gene copy-number variation analysis of *SOX2* and *SOX4* in bladder tumors from TCGA_BLCA (cBioPortal) cohort.

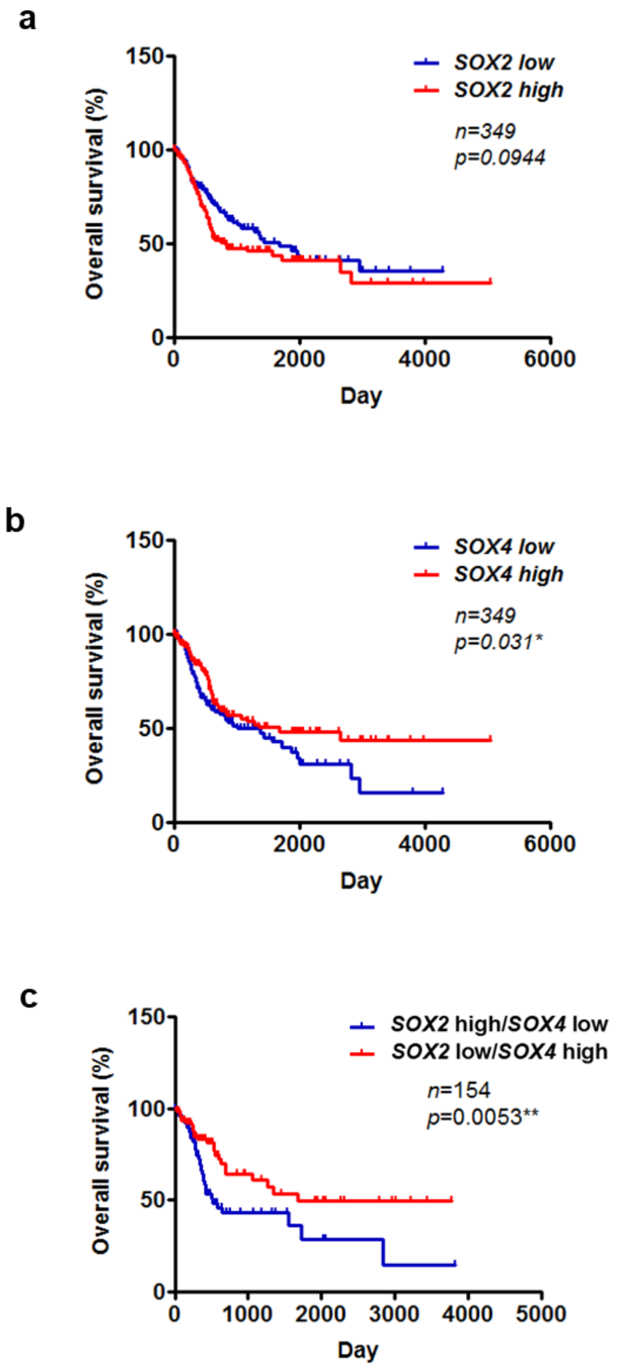

Figure S7. *SOX4* signifies a good survival in bladder cancer patients.

(a-b) Kaplan–Meier analysis of *SOX2* (a) and *SOX4* (b) expression with overall survival in patients from TCGA_BLCA cohort. The significance was examined by log-rank test. (c) Kaplan–Meier analysis to assess the correlation of *SOX2*-high/*SOX4*-low and *SOX2*-low/*SOX4*-high signatures with overall survival in patients from TCGA_BLCA cohort. The significance was examined by log-rank test.

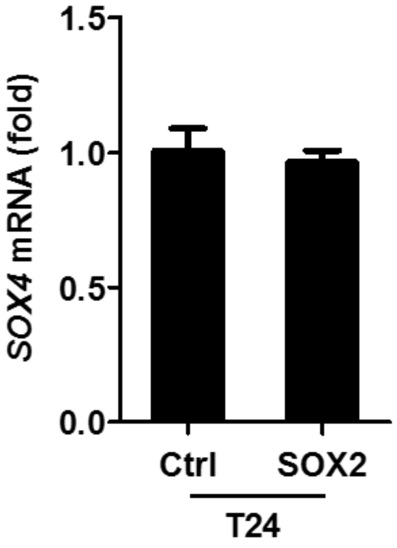

Figure S8. *SOX4* expression is not affected by *SOX2* expression in bladder cancer cells.

qPCR analysis to assess *SOX4* expression in T24 cells transduced with the lentiviral vector encoding *SOX2* cDNA (SOX2) or empty control vector (Ctrl). Results are the average of three replicates and expressed as the mean ± S.D.

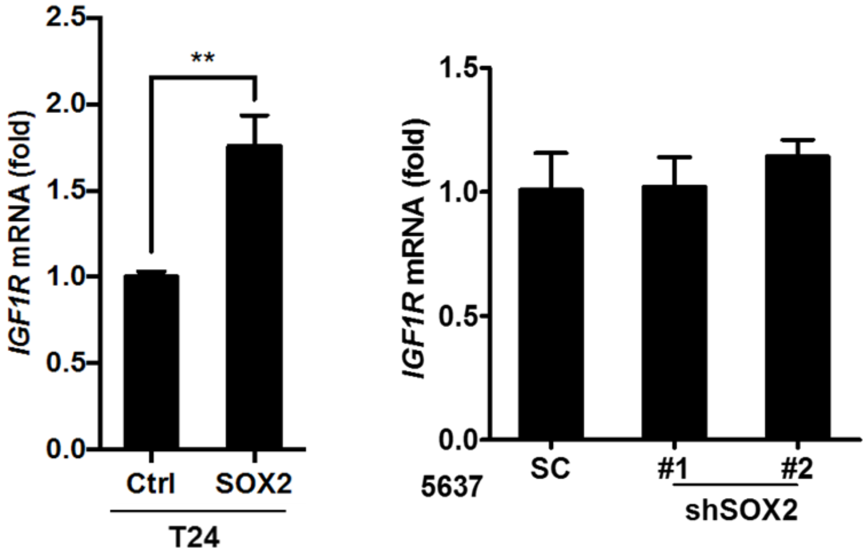

Figure S9. Effect of *SOX2* expression on *IGF1R* expression.

qPCR analysis to assess *IGF1R* expression in SOX2-expressing T24 cells (left) and SOX2-silenced 5637 cells (right). Results are the average of three replicates and expressed as the mean ± S.D. $^{**}P < 0.01$.

Table S2. Primer and probe sequence list

Gene	Forward primer sequence (5' to 3')	Reverse primer sequence (5' to 3')	Probe
18S	TGGCTCATTAAATCA GTTATG	CGGCATGATATTAGC TCTA	FAM-CGCTCGCT CCTCTCCTACTT G-BHQ1
SOX2	GCAGTACAACTCCAT GAC	GAGGAAGAGGTAAC CACA	FAM-CGCAGAC CTACATGAACG GC-BHQ
IGF2	CCAGAAGAGCTTGT CAGCAA	AGGACCTGCCATCAT ACTGC	UPL#52
IGF1R	TTCAGCGCTGCTGAT GTG	AAGTTCCCGGCTCAT GGT	UPL#7
IGFBP1	AATGGATTTTATCAC AGCAGACAG	GGTAGACGCACCAG CAGAGT	UPL#58
IGF2 (ChIP)	GCGTCTGCCATGACT CCT	GAGACCGCGAGAGG AAGAC	UPL#58

Table S3. The clinicopathologic information of bladder cancer tissues from BLC661 tissue array (US BIOMAX)

Case no.	Sex	Age	Organ	Pathology diagnosis	Type	Grade	TMN	IHC score			Expression
								PS	IS	Sum	
1	M	27	Bladder	TCC	Malignant	I	TisN0M0	4	2	6	Positive
2	M	50	Bladder	TCC	Malignant	I	T1N0M0	0	0	0	Negative
3	F	49	Bladder	TCC	Malignant	I	T1N0M0	0	0	0	Negative
4	M	67	Bladder	TCC	Malignant	I	T1N0M0	0	0	0	Negative
5	F	51	Bladder	TCC	Malignant	II	T1N0M0	2	1	3	Positive
6	M	57	Bladder	TCC	Malignant	I	T1N0M0	0	0	0	Negative
7	M	47	Bladder	TCC	Malignant	I	T2N0M0	0	0	0	Negative
8	M	54	Bladder	TCC	Malignant	II	T2N0M0	0	0	0	Negative
9	M	45	Bladder	TCC	Malignant	II	T1N0M0	0	0	0	Negative
10	M	74	Bladder	TCC	Malignant	II	T2N0M0	2	2	4	Positive
11	M	51	Bladder	TCC	Malignant	II	T1N0M0	2	1	3	Positive
12	M	80	Bladder	TCC	Malignant	II	T2N0M0	1	1	2	Positive
13	F	53	Bladder	TCC	Malignant	I	T1N0M0	0	0	0	Negative
14	M	37	Bladder	TCC	Malignant	III	T2N0M0	3	3	6	Positive
15	M	55	Bladder	TCC	Malignant	I	T4N2MX	0	0	0	Negative
16	M	52	Bladder	TCC	Malignant	II	T1N0M0	0	0	0	Negative
17	M	78	Bladder	TCC	Malignant	II	T1N0M0	0	0	0	Negative
18	M	64	Bladder	TCC	Malignant	III	T3N2M1	4	3	7	Positive
19	M	70	Bladder	TCC	Malignant	III	T2N0M0	3	2	5	Positive
20	M	61	Bladder	TCC	Malignant	III	T2N0M0	4	2	6	Positive
21	M	61	Bladder	TCC	Malignant	III	T1N0M0	3	2	5	Positive
22	F	39	Bladder	TCC	Malignant	III	T2N0M0	0	0	0	Negative

TCC: Transitional cell carcinoma
PS: Percentage score (0-5)
IS: Intensity score (0-3)

Table S4. Public domain data used in this study

Dataset	Patient Number	Clinical Outcome	PMID	Source [reference]
TCGA_BLCA	279	OS<br>RFS		UCSC cancer browser [1]
GSE32894	308	DFS<br>Grade	22553347	GEO browser [2]
GSE73211		gene expression	26501951	GEO browser [2]
ENCODE		H3K4me3<br>epigenetic regulation	22955616	ENCODE [3]
cBioPortal	127	gene alternation	22588877<br>23550210	cBioProtal [4]

[1] https://genome.ucsc.edu/
[2] http://www.ncbi.nlm.nih.gov/geo/query/acc.cgi?acc=GSE32894
[3] https://www.encodeproject.org
[4] http://www.cbioportal.org

Table S5. Spheroid analysis of three independent assays in Figure 3b

T24	Day1		Day17	
	Ctrl (0.1 mm^3)	SOX2 (0.1 mm^3)	Ctrl (0.1 mm^3)	SOX2 (0.1 mm^3)
1st	3.51	3.08	1.16	2.00
	3.06	3.03	1.01	2.57
	4.18	2.69	0.81	2.31
2nd	2.92	3.51	0.48	2.97
	2.75	3.17	0.48	2.52
	3.51	3.32	0.49	3.01
3rd	4.43	4.23	1.56	2.62
	4.00	4.44	2.05	3.01
	4.50	3.39	2.34	3.14

Table S6. 3D colony-forming analysis of three independent assays in Figure 3f and 5f

	T24-SOX2 (μm)		
**MK2206**	**-**	**+**	**-**
**Linsitinib**	**-**	**-**	**+**
1st	80.60	35.74	61.85
	73.92	34.01	48.69
	69.98	40.86	41.78
2nd	69.86	40.66	31.09
	68.56	43.69	31.87
	67.31	38.35	36.29
3rd	59.28	33.24	29.77
	48.06	28.32	31.28
	46.54	35.85	29.95

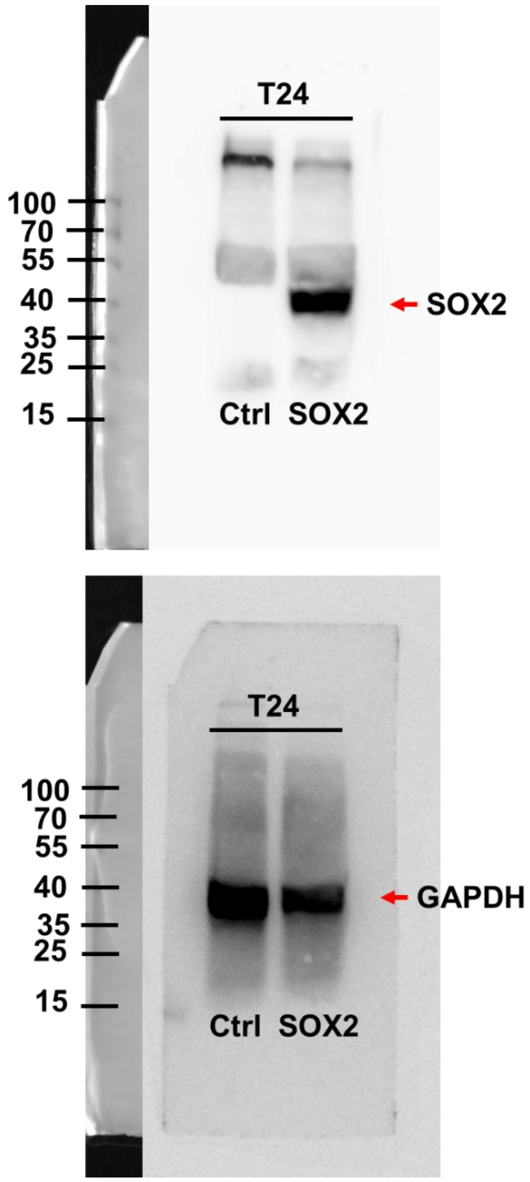

Figure S10. The images of full-length blots of western analysis performed on *SOX2*-expressing (SOX2) and control (Ctrl) T24 cells in Figure 2a.

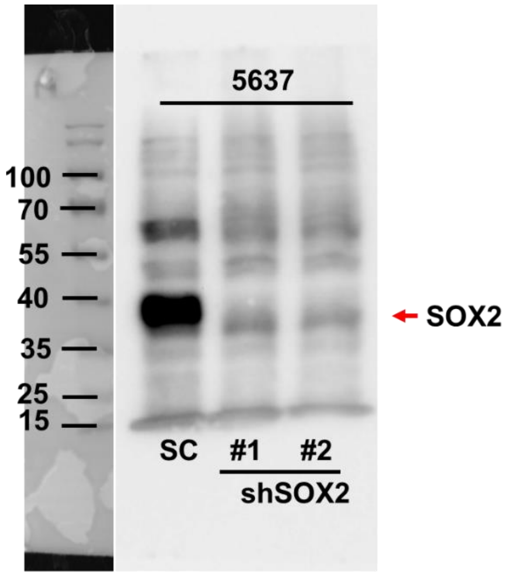

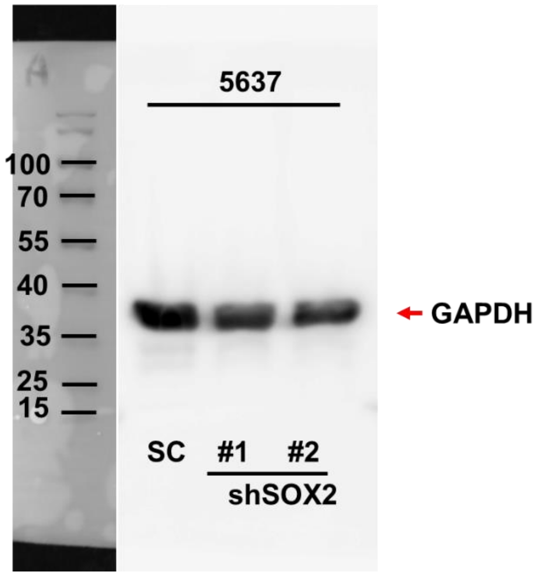

Figure S11. The images of full-length blots of western analysis performed on 5637 cells transduced with shSOX2 or scrambled control (SC) vector in Figure 2b.

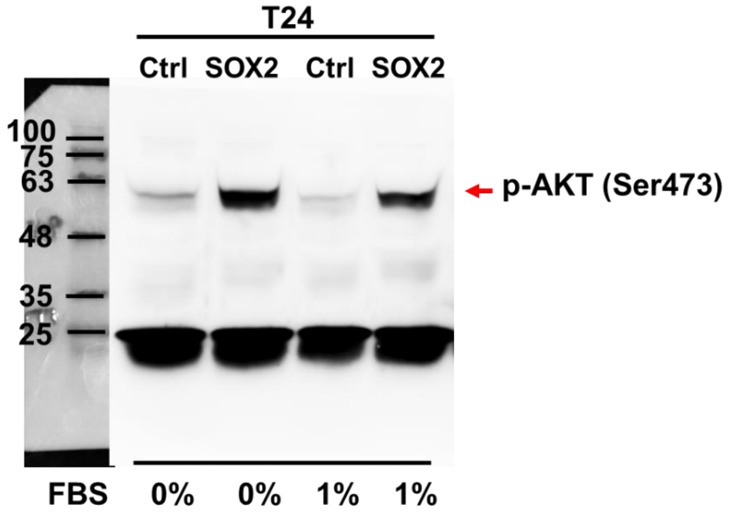

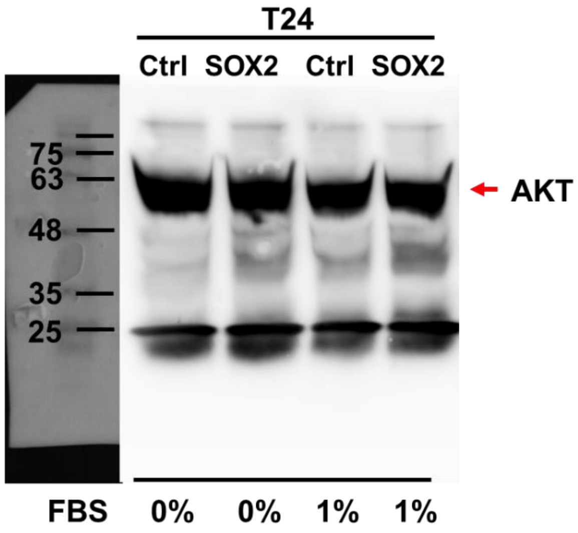

Figure S12. The images of full-length blots of western analysis performed on *SOX2*-expresing (SOX2) and control (Ctrl) T24 cells in Figure 3d.

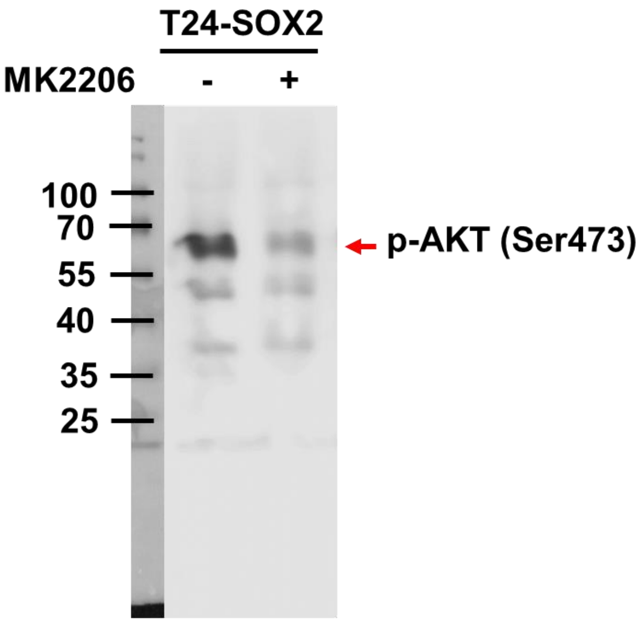

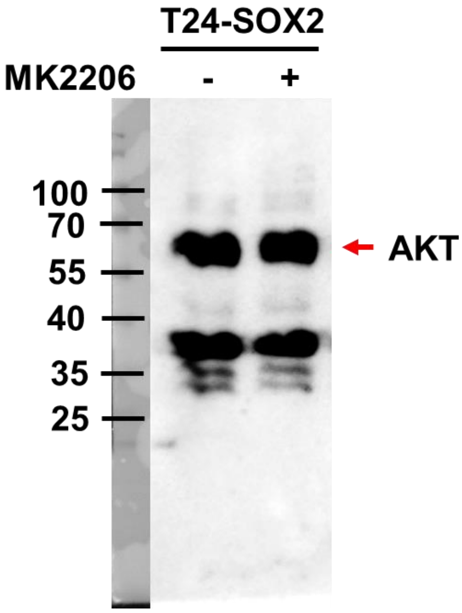

Figure S13. The images of full-length blots of western analysis performed on *SOX2*-expresing T24 cells in Figure 3e.

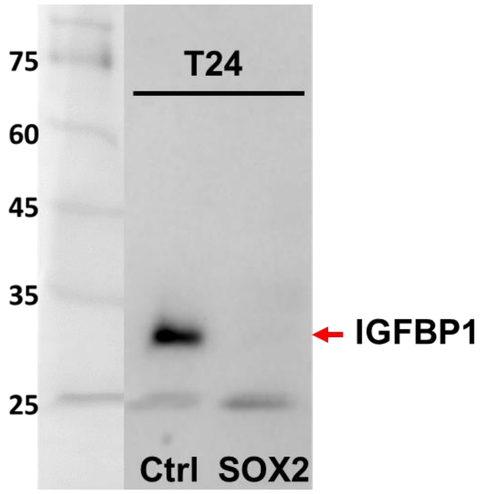

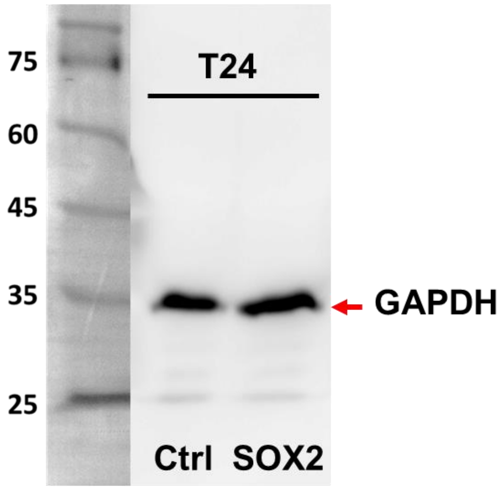

Figure S14. The images of full-length blots of western analysis performed on SOX2-expressing (SOX2) and control (Ctrl) T24 cells in Figure 4e (left).

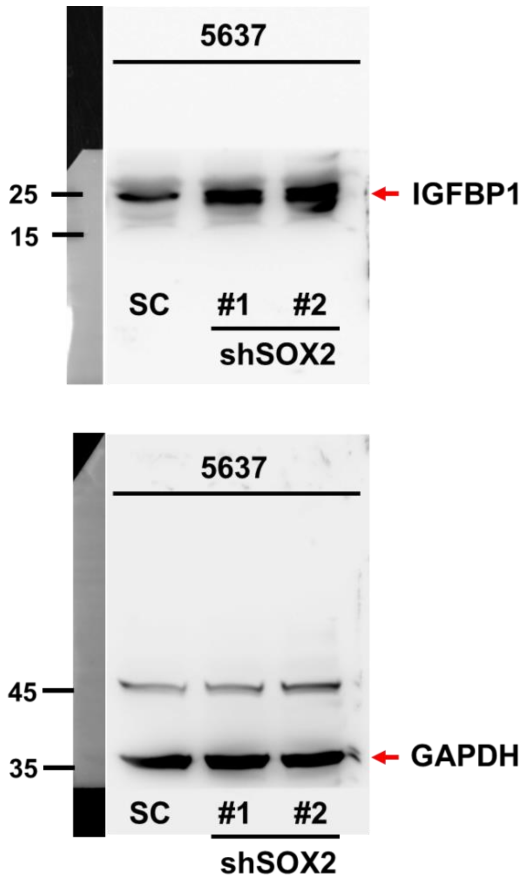

Figure S15. The images of full-length blots of western analysis performed on 5637 cells transduced with shSOX2 or scrambled control (SC) vector in Figure 4e (right).

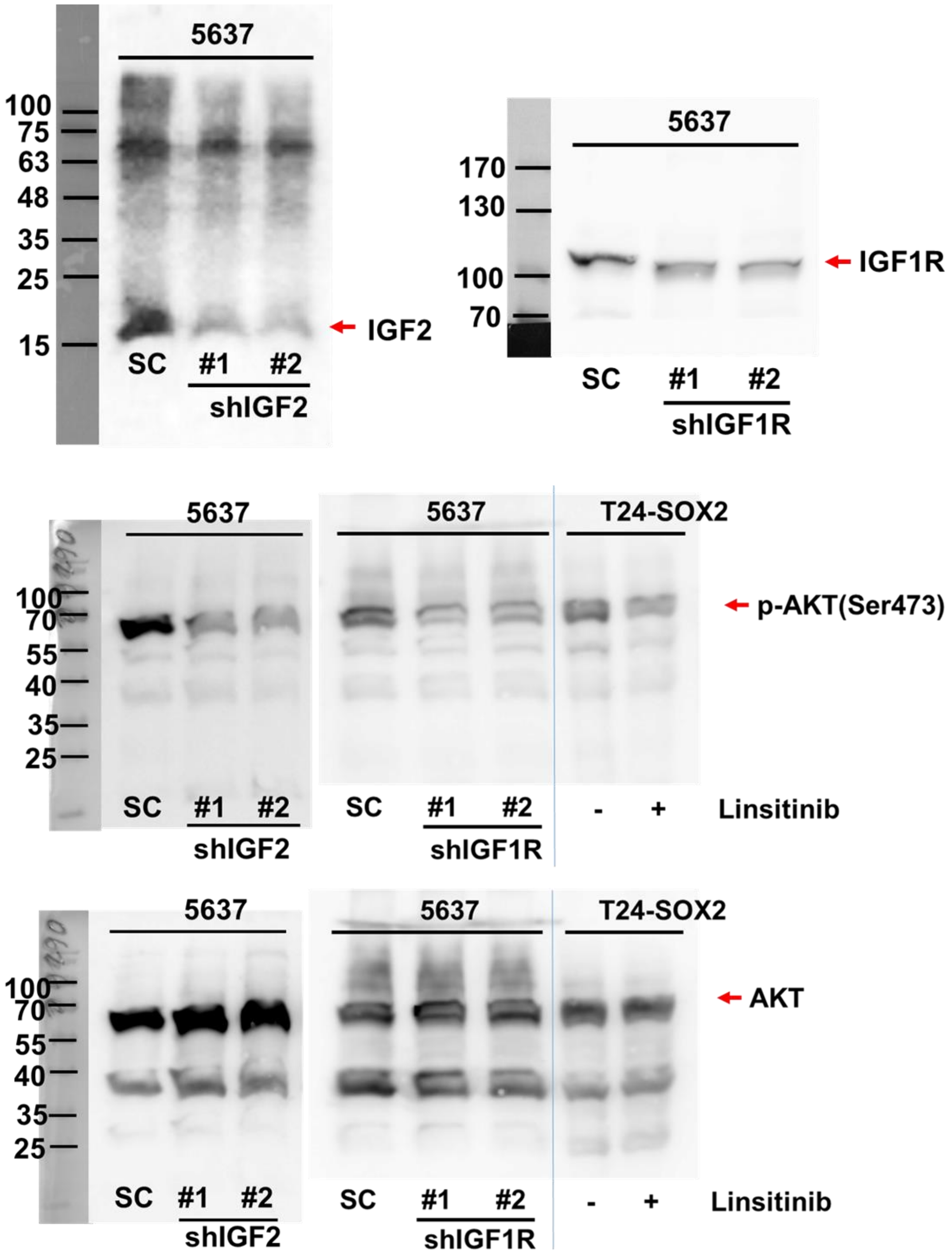

Figure S16. The images of full-length blots of western analysis performed on multiple cell lines presented in Figure 5a and 5c.

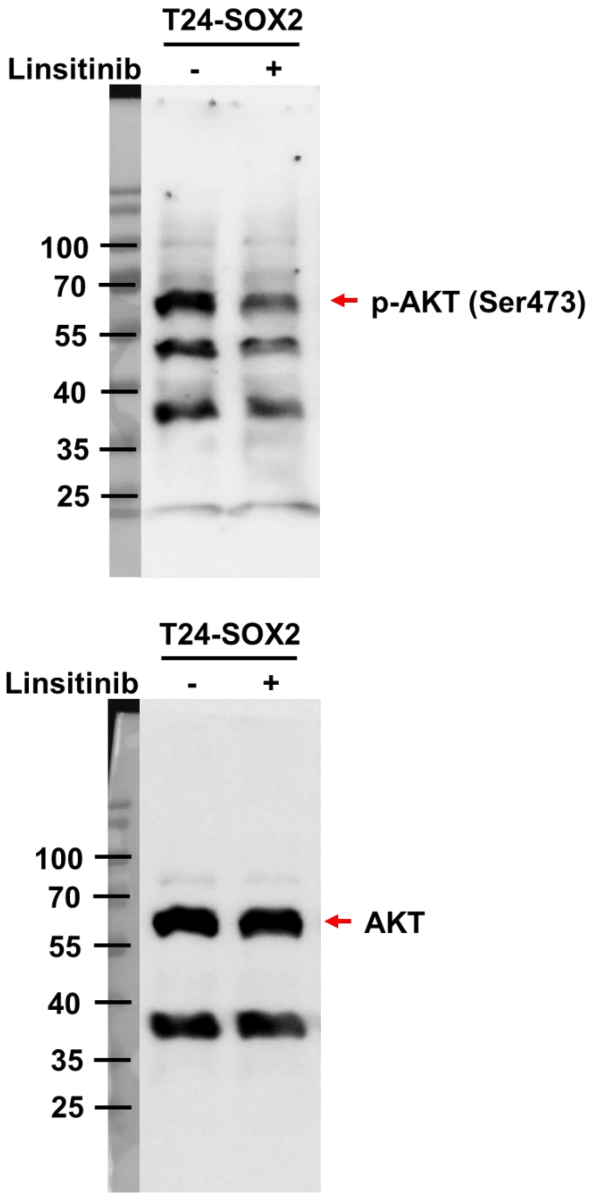

Figure S17. The images of full-length blots of western analysis performed on *SOX2*-expressing T24 cells presented in the Figure 5e.
